# Supplementary material for: Tozinameran (Pfizer, BioNTech) and Elasomeran (Moderna) Efficacy in COVID-19—A Systematic Review of Randomised Controlled Trial Studies
Source: Healthcare (Basel). 2023 May 24;11(11):1532. doi: 10.3390/healthcare11111532 (PMC10252750; doi:10.3390/healthcare11111532)
Supplement: Supplementary file 1 [file healthcare-11-01532-s001.zip › healthcare-2298348-supplementary.pdf]

Table S1. MEDLINE via PubMed search result.

No.	Keyword	Results
#1	Vaccine, BNT162 OR COVID-19 Vaccine Pfizer-BioNTech OR COVID 19 Vaccine Pfizer BioNTech OR Pfizer-BioNTech, COVID-19 Vaccine OR Vaccine Pfizer-BioNTech, COVID-19 OR Pidacmeran OR BNT-162C2 OR BNT 162C2 OR BNT162C2 OR Abdavomeran OR BNT-162B1 OR BNT 162B1 OR BNT162B1 OR BNT-162A1 OR BNT 162A1 OR BNT162A1 OR Tozinameran OR Pfizer Covid-19 Vaccine OR Covid-19 Vaccine, Pfizer OR Pfizer Covid 19 Vaccine OR Vaccine, Pfizer Covid-19 OR Comirnaty OR BNT162B2 OR BNT-162B2 OR BNT 162B2	3582
#2	Random* OR randomised OR randomized OR randomly OR randomisation OR randomization OR RCT OR controlled OR control OR controls OR prospective OR prospectived OR prospectives OR prospectively OR comparative OR randomized OR comparatively OR study OR studies OR cohort OR cohorts OR retrospective OR randomized OR retrospectives OR retrospectively OR observational OR experimental OR cross-sectional OR cross sectional OR follow up OR follow-up OR trial OR trail OR trials OR blind OR blinded OR blinding OR mask OR masked OR masking OR Clinical Trials, Randomized OR Trials, Randomized Clinical OR Controlled Clinical Trials, Randomized	18046023
#3	2019 nCoV Vaccine mRNA 1273 OR mRNA-1273, 2019-nCoV Vaccine OR Moderna COVID-19 Vaccine OR COVID-19 Vaccine, Moderna OR Moderna COVID 19 Vaccine OR Vaccine, Moderna COVID-19 OR Elasomeran OR Moderna COVID-19 Vaccine RNA OR Moderna COVID 19 Vaccine RNA OR COVID-19 Vaccine Moderna OR COVID 19 Vaccine Moderna OR Moderna, COVID-19 Vaccine OR mRNA-1273 OR mRNA 1273 OR TAK-919 OR TAK 919 OR TAK919 OR M-1273 OR M 1273 OR M1273 OR mRNA-1273.211 OR mRNA 1273.211	1669
#4	Humans OR Homo sapiens OR Man (Taxonomy) OR Man, Modern OR Modern Man OR Human OR Man OR Patient OR Patients OR Client OR Clients	21980155
#5	COVID 19 Vaccines OR Vaccines, COVID-19 OR COVID-19 Virus Vaccines OR COVID 19 Virus Vaccines OR Vaccines, COVID-19 Virus OR Virus Vaccines, COVID-19 OR COVID-19 Virus Vaccine OR COVID 19 Virus Vaccine OR Vaccine, COVID-19 Virus OR Virus Vaccine, COVID-19 OR COVID19 Virus Vaccines OR Vaccines, COVID19 Virus OR Virus Vaccines, COVID19 OR COVID19 Virus Vaccine OR Vaccine, COVID19 Virus OR Virus Vaccine, COVID19 OR COVID19 Vaccines OR Vaccines, COVID19 OR COVID19 Vaccine OR Vaccine, COVID19 OR SARS-CoV-2 Vaccines OR SARS CoV 2 Vaccines OR Vaccines, SARS-CoV-2 OR SARS-CoV-2 Vaccine OR SARS CoV 2 Vaccine OR Vaccine, SARS-CoV-2 OR SARS2 Vaccines OR Vaccines, SARS2 OR SARS2 Vaccine OR Vaccine, SARS2 OR Coronavirus Disease 2019 Vaccines OR Coronavirus Disease 2019 Vaccine OR Coronavirus Disease 2019 Virus Vaccine OR Coronavirus Disease 2019 Virus Vaccines	21836

No.	Keyword	Results
	OR Coronavirus Disease-19 Vaccines OR Coronavirus Disease 19 Vaccines OR Vaccines, Coronavirus Disease-19 OR Coronavirus Disease-19 Vaccine OR Coronavirus Disease 19 Vaccine OR Vaccine, Coronavirus Disease-19 OR COVID 19 Vaccine OR Vaccine, COVID 19 OR 2019-nCoV Vaccine OR 2019 nCoV Vaccine OR Vaccine, 2019-nCoV OR 2019 Novel Coronavirus Vaccines OR 2019 Novel Coronavirus Vaccine OR 2019-nCoV Vaccines OR 2019 nCoV Vaccines OR Vaccines, 2019-nCoV OR COVID-19 Vaccine OR Vaccine, COVID-19 OR SARS Coronavirus 2 Vaccines	
#6	#1 AND #2 AND #4 AND #5	1786
#7	#2 AND #3 AND #4 AND #5	692
#8	#6 AND #7	1993

Table S1A. Additional keywords used for data presentation in Table. 1 (manuscript). Tozinameran and elasomeran data in Medline via PubMed (2020 - 2022) .

Adverse events	Drug Related Side Effects and Adverse Reactions OR Drug-Related Side Effects and Adverse Reaction OR Drug Related Side Effects and Adverse Reaction OR Drug Side Effects OR Drug Side Effect OR Effects, Drug Side OR Side Effect, Drug OR Side Effects, Drug OR Adverse Drug Reaction OR Adverse Drug Reactions OR Drug Reaction, Adverse OR Drug Reactions, Adverse OR Reactions, Adverse Drug OR Adverse Drug Event OR Adverse Drug Events OR Drug Event, Adverse OR Drug Events, Adverse OR Side Effects of Drugs OR Drug Toxicity OR Toxicity, Drug OR Drug Toxicities OR Toxicities, Drug
Outcome (effectiveness, efficacy)	Outcome, Treatment OR Patient-Relevant Outcome OR Outcome, Patient-Relevant OR Outcomes, Patient-Relevant OR Patient Relevant Outcome OR Patient-Relevant Outcomes OR Clinical Effectiveness OR Effectiveness, Clinical OR Treatment Effectiveness OR Effectiveness, Treatment OR Rehabilitation Outcome OR Outcome, Rehabilitation OR Treatment Efficacy OR Efficacy, Treatment OR Clinical Efficacy OR Efficacy, Clinical

Table S2. EMBASE search result.

No.	Keywords	Results
#1	'tozinameran'/exp OR tozinameran OR 'biontech covid-19 vaccine'/exp OR 'biontech covid-19 vaccine' OR (('biontech'/exp OR biontech) AND ('covid 19'/exp OR 'covid 19') AND ('vaccine'/exp OR vaccine)) OR 'biontech-pfizer covid-19 vaccine'/exp OR 'biontech-pfizer covid-19 vaccine' OR ('biontech pfizer' AND ('covid 19'/exp OR 'covid 19') AND ('vaccine'/exp OR vaccine)) OR 'bnt 162b2'/exp OR 'bnt 162b2' OR (bnt AND 162b2) OR 'bnt162b2'/exp OR bnt162b2 OR 'comirnaty'/exp OR comirnaty OR 'covid-19 vaccine biontech'/exp OR 'covid-19 vaccine biontech' OR (('covid 19'/exp OR 'covid 19') AND ('vaccine'/exp OR vaccine) AND ('biontech'/exp OR biontech)) OR 'covid-19 vaccine biontech-pfizer'/exp OR 'covid-19 vaccine biontech-pfizer' OR (('covid 19'/exp OR 'covid 19') AND ('vaccine'/exp OR vaccine) AND 'biontech pfizer') OR 'covid-19 vaccine pfizer'/exp OR 'covid-19 vaccine pfizer' OR (('covid 19'/exp OR 'covid 19') AND ('vaccine'/exp OR vaccine) AND ('pfizer'/exp OR pfizer)) OR 'covid-19 vaccine pfizer-biontech'/exp OR 'covid-19 vaccine pfizer-biontech' OR (('covid 19'/exp OR 'covid 19') AND ('vaccine'/exp OR vaccine) AND ('pfizer biontech'/exp OR 'pfizer biontech')) OR 'pf 07302048'/exp OR 'pf 07302048' OR (pf AND 07302048) OR 'pf07302048'/exp OR pf07302048 OR 'pfizer covid-19 vaccine'/exp OR 'pfizer covid-19 vaccine' OR (('pfizer'/exp OR pfizer) AND ('covid 19'/exp OR 'covid 19') AND ('vaccine'/exp OR vaccine)) OR 'pfizer-biontech covid-19 vaccine'/exp OR 'pfizer-biontech covid-19 vaccine' OR (('pfizer biontech'/exp OR 'pfizer biontech') AND ('covid 19'/exp OR 'covid 19') AND ('vaccine'/exp OR vaccine))	5034
#2	'elasomeran'/exp OR elasomeran OR '2019-ncov vaccine mrna-1273'/exp OR '2019-ncov vaccine mrna-1273' OR (('2019 ncov'/exp OR '2019 ncov') AND ('vaccine'/exp OR vaccine) AND ('mrna 1273'/exp OR 'mrna 1273')) OR '2019-ncov vaccine mrna1273'/exp OR '2019-ncov vaccine mrna1273' OR (('2019 ncov'/exp OR '2019 ncov') AND ('vaccine'/exp OR vaccine) AND ('mrna1273'/exp OR mrna1273)) OR 'covid-19 vaccine moderna'/exp OR 'covid-19 vaccine moderna' OR (('covid 19'/exp OR 'covid 19') AND ('vaccine'/exp OR vaccine) AND ('moderna'/exp OR moderna)) OR 'covid-19 vaccine mrna-1273'/exp OR 'covid-19 vaccine mrna-1273' OR (('covid 19'/exp OR 'covid 19') AND ('vaccine'/exp OR vaccine) AND ('mrna 1273'/exp OR 'mrna 1273')) OR 'covid-19 vaccine mrna1273'/exp OR 'covid-19 vaccine mrna1273' OR (('covid 19'/exp OR 'covid 19') AND ('vaccine'/exp OR vaccine) AND ('mrna1273'/exp OR mrna1273)) OR 'cx 024414'/exp OR 'cx 024414' OR (cx AND 024414) OR 'cx024414'/exp OR cx024414 OR 'messenger rna-1273'/exp OR 'messenger rna-1273' OR (messenger AND 'rna 1273') OR 'messenger rna-1273 vaccine'/exp OR 'messenger rna-1273 vaccine' OR (messenger AND 'rna 1273' AND ('vaccine'/exp OR vaccine)) OR 'messenger rna1273'/exp OR 'messenger rna1273' OR (messenger AND rna1273) OR 'messenger rna1273 vaccine'/exp OR 'messenger rna1273 vaccine' OR (messenger AND rna1273 AND ('vaccine'/exp OR vaccine)) OR 'moderna covid-19 vaccine'/exp OR 'moderna covid-19 vaccine'	2733

	OR (('moderna'/exp OR moderna) AND ('covid 19'/exp OR 'covid 19') AND ('vaccine'/exp OR vaccine)) OR 'mrna 1273'/exp OR 'mrna 1273' OR 'mrna-1273 vaccine'/exp OR 'mrna-1273 vaccine' OR (('mrna 1273'/exp OR 'mrna 1273') AND ('vaccine'/exp OR vaccine)) OR 'mrna1273'/exp OR mrna1273 OR 'mrna1273 vaccine'/exp OR 'mrna1273 vaccine' OR (('mrna1273'/exp OR mrna1273) AND ('vaccine'/exp OR vaccine)) OR 'spikevax'/exp OR spikevax OR 'tak 919'/exp OR 'tak 919' OR (tak AND 919) OR 'tak919'/exp OR tak919	
3#	'controlled trial, randomized'/exp OR 'controlled trial, randomized' OR (controlled AND trial, AND randomized) OR 'randomised controlled study'/exp OR 'randomised controlled study' OR (randomised AND controlled AND ('study'/exp OR study)) OR 'randomised controlled trial'/exp OR 'randomised controlled trial' OR (randomised AND controlled AND ('trial'/exp OR trial)) OR 'randomized controlled study'/exp OR 'randomized controlled study' OR (randomized AND controlled AND ('study'/exp OR study)) OR 'trial, randomized controlled'/exp OR 'trial, randomized controlled' OR (trial, AND randomized AND controlled) OR 'random allocation'/exp OR 'random allocation' OR (random AND allocation) OR 'randomisation'/exp OR randomisation OR 'double blind clinical trial'/exp OR 'double blind clinical trial' OR (double AND ('blind'/exp OR blind) AND ('clinical'/exp OR clinical) AND ('trial'/exp OR trial)) OR 'double blind comparison'/exp OR 'double blind comparison' OR (double AND ('blind'/exp OR blind) AND ('comparison'/exp OR comparison)) OR 'double blind design'/exp OR 'double blind design' OR (double AND ('blind'/exp OR blind) AND ('design'/exp OR design)) OR 'double blind studies'/exp OR 'double blind studies' OR (double AND ('blind'/exp OR blind) AND ('studies'/exp OR studies)) OR 'double blind study'/exp OR 'double blind study' OR (double AND ('blind'/exp OR blind) AND ('study'/exp OR study)) OR 'double blind test'/exp OR 'double blind test' OR (double AND ('blind'/exp OR blind) AND ('test'/exp OR test)) OR 'double blind trial'/exp OR 'double blind trial' OR (double AND ('blind'/exp OR blind) AND ('trial'/exp OR trial)) OR 'double masked clinical study'/exp OR 'double masked clinical study' OR (double AND masked AND ('clinical'/exp OR clinical) AND ('study'/exp OR study)) OR 'double masked clinical trial'/exp OR 'double masked clinical trial' OR (double AND masked AND ('clinical'/exp OR clinical) AND ('trial'/exp OR trial)) OR 'double masked comparison'/exp OR 'double masked comparison' OR (double AND masked AND ('comparison'/exp OR comparison)) OR 'double masked design'/exp OR 'double masked design' OR (double AND masked AND ('design'/exp OR design)) OR 'double masked method'/exp OR 'double masked method' OR (double AND masked AND ('method'/exp OR method)) OR 'double masked procedurę' OR (double AND masked AND procedurę) OR 'double masked study'/exp OR 'double masked study' OR (double AND masked AND ('study'/exp OR study)) OR 'double masked test'/exp OR 'double masked test' OR (double AND masked AND ('test'/exp OR test)) OR 'double masked trial'/exp OR 'double masked trial' OR (double AND masked AND ('trial'/exp OR trial)) OR 'double-blind clinical study'/exp OR 'double-blind clinical study' OR ('double blind' AND ('clinical'/exp OR clinical) AND ('study'/exp OR study)) OR 'double-blind method'/exp OR 'double-blind method' OR ('double blind' AND ('method'/exp OR method))	1252648

#4	'sars-cov-2 vaccine'/exp OR 'sars-cov-2 vaccine' OR (('sars cov 2'/exp OR 'sars cov 2') AND ('vaccine'/exp OR vaccine)) OR '2019-ncov vaccine'/exp OR '2019-ncov vaccine' OR (('2019 ncov'/exp OR '2019 ncov') AND ('vaccine'/exp OR vaccine)) OR '2019-ncov virus vaccine'/exp OR '2019-ncov virus vaccine' OR (('2019 ncov'/exp OR '2019 ncov') AND ('virus'/exp OR virus) AND ('vaccine'/exp OR vaccine)) OR 'coronavirus disease 2019 vaccine'/exp OR 'coronavirus disease 2019 vaccine' OR (('coronavirus'/exp OR coronavirus) AND ('disease'/exp OR disease) AND 2019 AND ('vaccine'/exp OR vaccine)) OR 'covid 19 vaccine'/exp OR 'covid 19 vaccine' OR (('covid'/exp OR covid) AND 19 AND ('vaccine'/exp OR vaccine)) OR 'covid-19 mrna vaccine'/exp OR 'covid-19 mrna vaccine' OR (('covid 19'/exp OR 'covid 19') AND ('mrna'/exp OR mrna) AND ('vaccine'/exp OR vaccine)) OR 'covid-19 recombinant protein vaccine'/exp OR 'covid-19 recombinant protein vaccine' OR (('covid 19'/exp OR 'covid 19') AND ('recombinant'/exp OR recombinant) AND ('protein'/exp OR protein) AND ('vaccine'/exp OR vaccine)) OR 'covid-19 vaccine'/exp OR 'covid-19 vaccine' OR (('covid 19'/exp OR 'covid 19') AND ('vaccine'/exp OR vaccine)) OR 'covid-19 vaccines'/exp OR 'covid-19 vaccines' OR (('covid 19'/exp OR 'covid 19') AND ('vaccines'/exp OR vaccines)) OR 'covid-19 virus vaccine'/exp OR 'covid-19 virus vaccine' OR (('covid 19'/exp OR 'covid 19') AND ('virus'/exp OR virus) AND ('vaccine'/exp OR vaccine)) OR 'covid19 vaccine'/exp OR 'covid19 vaccine' OR (('covid19'/exp OR covid19) AND ('vaccine'/exp OR vaccine)) OR 'covid19 virus vaccine'/exp OR 'covid19 virus vaccine' OR (('covid19'/exp OR covid19) AND ('virus'/exp OR virus) AND ('vaccine'/exp OR vaccine)) OR 'hcov-19 vaccine'/exp OR 'hcov-19 vaccine' OR (('hcov 19'/exp OR 'hcov 19') AND ('vaccine'/exp OR vaccine)) OR 'hcov-19 virus vaccine'/exp OR 'hcov-19 virus vaccine' OR (('hcov 19'/exp OR 'hcov 19') AND ('virus'/exp OR virus) AND ('vaccine'/exp OR vaccine)) OR 'human coronavirus 2019 vaccine'/exp OR 'human coronavirus 2019 vaccine' OR (('human'/exp OR human) AND ('coronavirus'/exp OR coronavirus) AND 2019 AND ('vaccine'/exp OR vaccine)) OR 'inactivated covid-19 vaccine'/exp OR 'inactivated covid-19 vaccine' OR (inactivated AND ('covid 19'/exp OR 'covid 19') AND ('vaccine'/exp OR vaccine)) OR 'inactivated sars-cov-2 vaccine'/exp OR 'inactivated sars-cov-2 vaccine' OR (inactivated AND ('sars cov 2'/exp OR 'sars cov 2') AND ('vaccine'/exp OR vaccine)) OR 'mrna covid-19 vaccine'/exp OR 'mrna covid-19 vaccine' OR (('mrna'/exp OR mrna) AND ('covid 19'/exp OR 'covid 19') AND ('vaccine'/exp OR vaccine)) OR 'mrna sars-cov-2 vaccine'/exp OR 'mrna sars-cov-2 vaccine' OR (('mrna'/exp OR mrna) AND ('sars cov 2'/exp OR 'sars cov 2') AND ('vaccine'/exp OR vaccine)) OR 'ncov-2019 vaccine'/exp OR 'ncov-2019 vaccine' OR (('ncov 2019'/exp OR 'ncov 2019') AND ('vaccine'/exp OR vaccine)) OR 'ncov-2019 virus vaccine'/exp OR 'ncov-2019 virus vaccine' OR (('ncov 2019'/exp OR 'ncov 2019') AND ('virus'/exp OR virus) AND ('vaccine'/exp OR vaccine)) OR 'novel 2019 coronavirus vaccine'/exp OR 'novel 2019 coronavirus vaccine' OR (novel AND 2019 AND ('coronavirus'/exp OR coronavirus) AND ('vaccine'/exp OR vaccine)) OR 'novel coronavirus 2019 vaccine'/exp OR 'novel coronavirus 2019 vaccine' OR (novel AND ('coronavirus'/exp OR coronavirus) AND 2019 AND ('vaccine'/exp OR vaccine)) OR 'sars coronavirus 2 vaccine'/exp OR 'sars coronavirus 2 vaccine' OR (('sars'/exp OR sars) AND ('coronavirus'/exp OR coronavirus)	30644

	AND ('2'/exp OR 2) AND ('vaccine'/exp OR vaccine)) OR 'sars-cov-2 inactivated vaccine'/exp OR 'sars-cov-2 inactivated vaccine' OR (('sars cov 2'/exp OR 'sars cov 2') AND inactivated AND ('vaccine'/exp OR vaccine)) OR 'sars-cov-2 mrna vaccine'/exp OR 'sars-cov-2 mrna vaccine' OR (('sars cov 2'/exp OR 'sars cov 2') AND ('mrna'/exp OR mrna) AND ('vaccine'/exp OR vaccine)) OR 'sars-cov-2 recombinant protein vaccine'/exp OR 'sars-cov-2 recombinant protein vaccine' OR (('sars cov 2'/exp OR 'sars cov 2') AND ('recombinant'/exp OR recombinant) AND ('protein'/exp OR protein) AND ('vaccine'/exp OR vaccine)) OR 'sars-cov-2 virus vaccine'/exp OR 'sars-cov-2 virus vaccine' OR (('sars cov 2'/exp OR 'sars cov 2') AND ('virus'/exp OR virus) AND ('vaccine'/exp OR vaccine)) OR 'sars2 vaccine'/exp OR 'sars2 vaccine' OR (sars2 AND ('vaccine'/exp OR vaccine)) OR 'sars2 virus vaccine'/exp OR 'sars2 virus vaccine' OR (sars2 AND ('virus'/exp OR virus) AND ('vaccine'/exp OR vaccine)) OR 'severe acute respiratory syndrome 2 vaccine'/exp OR 'severe acute respiratory syndrome 2 vaccine' OR (severe AND acute AND ('respiratory'/exp OR respiratory) AND ('syndrome'/exp OR syndrome) AND ('2'/exp OR 2) AND ('vaccine'/exp OR vaccine)) OR 'severe acute respiratory syndrome coronavirus 2 vaccine'/exp OR 'severe acute respiratory syndrome coronavirus 2 vaccine' OR (severe AND acute AND ('respiratory'/exp OR respiratory) AND ('syndrome'/exp OR syndrome) AND ('coronavirus'/exp OR coronavirus) AND ('2'/exp OR 2) AND ('vaccine'/exp OR vaccine)) OR 'wuhan coronavirus vaccine'/exp OR 'wuhan coronavirus vaccine' OR (('wuhan'/exp OR wuhan) AND ('coronavirus'/exp OR coronavirus) AND ('vaccine'/exp OR vaccine))	
#5	#1 AND #3 AND #4	**178**
#6	#2 AND #3 AND #4	**123**
#7	#5 AND #6	**209**

Table S3. JADAD Scale.

Group of questions	Question	Score
basic	Is the study described as randomised?	0/1
	Is the study described as blind?	0/1
	Is information about the loss of patients from the study provided?	0/1
additional	Do you add one point for the given description of randomisation and the proper method?	+1
	Do you add one point for the given blinded description and the proper method?	+1
	Do you deduct one point for the incorrect randomisation method?	-1
	Do you subtract one point for the incorrect method of blinding?	-1

Table S4. Summary results of efficacy and safety of mRNA COVID-19 vaccines.

Publication	Type of the study	Credibility assessment	Vaccine	Number of doses, days	Study period	Population	Control intervention (comparator)	Method of administration	Research sponsor	Result – clinical response		Result - total adverse events (AE)		Result - total Serious Adverse Events (SAE)	
										mRNA	PBO	mRNA	PBO	mRNA	PBO
Haranaka et al. 2021	Phase I/II, randomised, Observer-blind	4.5/5	BNT162b2 (30 µg)	2 (0, 21)	21.10.2020-05.01.2021 77 days	20-85 y/o	placebo	intramuscularly	Pfizer/ BioNTech			12/119	3/41	0/119	0/41
Thomas et al. 2021	Phase I/II/III, randomised, observer-blind	4.5/5	BNT162b2 (30 µg)	2 (0,21)	27.07.2020-13.03.2021 230 days	≥16 y/o, healthy or controlled chronic diseases	placebo	intramuscularly	Pfizer/ BioNTech	77/ 20998	850/ 21096	6617/ 21926	3048/ 21921	127/ 21926	116/ 21921
Polack et al. 2020	Phase III, randomised, observer-blind, multicentre	4.5/5	BNT162b2 (30 µg)	2 (0, 21)	24.07.2020-09.10.2020 78 days	≥16 y/o, healthy or controlled chronic diseases	placebo	intramuscularly	Pfizer/ BioNTech	8/ 18198	162/ 18325	5770/ 21621	2638/ 21631	126/ 21621	111/ 21631
Walsh et al. 2020	Phase I, randomised, observer-blind, multicentre	4.5/5	BNT162b2 (30 µg)	2 (0, 21)	04.05.2020-22.06.2020 35 days	18-65 or ≥ 85 y/o, healthy	placebo	intramuscularly	Pfizer/ BioNTech			8/24	4/18	0/24	0/18

Publication	Type of the study	Credibility assessment	Vaccine	Number of doses, days	Study period	Population	Control intervention (comparator)	Method of administ ration	Research sponsor	Result – clinical response		Result - total adverse events (AE)		Result - total Serious Adverse Events (SAE)	
										mRNA	PBO	mRNA	PBO	mRNA	PBO
Chu et al. 2021	Phase II, randomised, observer-blind, multicentre	4.5/5	mRNA-1273 (100 µg)	2 (0, 28)	29.05.2020-08.07.2020 57 days	Zdrowi, 18-54 or ≥55 y/o, healthy	placebo	intramusc ularly	Moderna			Dose 1 176/200<br>Dose 2 178/198	Dose 1 77/200<br>Dose 2 66/194	0/200	0/200
Masuda et al. 2022	Phase I/II, randomised, Observer-blind, multicentre	4.5/5	mRNA-1273 (100 µg)	2 (0, 28)	21.01.2021-31.03.2021 70 days	18-64 or ≥ 65 y/o	placebo	intramusc ularly	Takeda Pharmaceut ical Company Limited			Dose 1 138/150<br>Dose 2 135/147	Dose 1 12/50<br>Dose 2 11/50	0/150	0/50
Sahly et al. 2021	Phase III, randomised, observer-blind, multicentre	4.5/5	mRNA-1273 (100 µg)	2 (0, 28)	27.07.2020-26.03.2020 243 days	18-64 or ≥65 y/o, with a higher risk of developing the disease	placebo	intramusc ularly	Moderna	55/ 14287	744/ 14164	Dose 1 13317/ 15166<br>Dose 2 13556/ 14691	Dose 1 7285/ 15151<br>Dose 2 6255/ 14578	401/151 84	439/ 15162
Baden et al. 2021	Phase III, randomised, observer-blind, multicentre	4.5/5	mRNA-1273 (100 µg)	2 (0, 28)	27.07.2020-21.11.2020 118 days	≥18 y/o, without a diagnosis of COVID-19, with an increased risk of developing the disease	placebo	intramusc ularly	Moderna	11/ 14134	185/ 14073	Dose 1 13319/ 15168<br>Dose 2 13534/ 14677	Dose 1 7284/ 15155<br>Dose 2 6232/ 14566	207/ 15185	211/ 15166

Table S5. JADAD score.

Question	Points	Haranaka et al. 2021	Thomas et al. 2021	Polack et al. 2020	Walsh et al. 2020	Chu et al. 2021	Masuda et al. 2022	Sahly et al. 2021	Baden et al. 2021
Is the study described as randomised?	0/1	1	1	1	1	1	1	1	1
Is the study described as blind?	0/1	0.5	0.5	0.5	0.5	0.5	0.5	0.5	0.5
Is information about the loss of patients from the study provided?	0/1	1	1	1	1	1	1	1	1
Do you add one point for the given description of randomisation and the proper method?	+1	1	1	1	1	1	1	1	1
Do you add one point for the given blinded description and the proper method?	+1	1	1	1	1	1	1	1	1
Do you deduct one point for the incorrect randomisation method?	-1	-	-	-	-	-	-	-	-
Do you subtract one point for the incorrect method of blinding?	-1	-	-	-	-	-	-	-	-
**Score:**	**-**	**4.5/5**	**4.5/5**	**4.5/5**	**4.5/5**	**4.5/5**	**4.5/5**	**4.5/5**	**4.5/5**

Table S6. Tozinameran GRADE assessment.

Certainty assessment							№ of patients		Effect		Certainty	Importance
**№ of studies**	**Study design**	**Risk of bias**	**Inconsistency**	**Indirectness**	**Imprecision**	**Other considerations**	**Tozinameran (BNT162b2)**	**Placebo**	**Relative (95% CI)**	**Absolute (95% CI)**		
**Clinical response - BNT162b2**												
2	randomised trials	not serious	serious[a]	not serious	not serious	very strong association	85/39196 (0.2%)	1012/39421 (2.6%)	**RR 0.08** (0.07 to 0.11)	**24 fewer per 1.000** (from 24 fewer to 23 fewer)	⨁⨁⨁⨁ High	CRITICAL
**AEs - BNT162b2**												
4	randomised trials	not serious	not serious	not serious	serious[b]	strong association	12407/43690 (28.4%)	5693/43611 (13.1%)	**RR 2.18** (2.12 to 2.24)	**154 more per 1.000** (from 146 more to 162 more)	⨁⨁⨁⨁ High	IMPORTANT
**SAEs - BNT162b2**												
4	randomised trials	not serious	not serious	not serious	serious[b]	none	253/43690 (0.6%)	227/43611 (0.5%)	**RR 1.11** (0.93 to 1.33)	**1 more per 1.000** (from 0 fewer to 2 more)	⨁⨁⨁◯ Moderate	CRITICAL

CI: confidence interval; **RR:** risk ratio

Explanations

a. High heterogeneity and p-value for heterogeneity; downgraded 1 steps for serious inconsistency

b. Wide confidence intervals and crossed threshold; downgraded 1 step for serious imprecision

Table S7. Elasomeran GRADE assessment.

Certainty assessment							№ of patients		Effect		Certainty	Importance
**№ of studies**	**Study design**	**Risk of bias**	**Inconsistency**	**Indirectness**	**Imprecision**	**Other considerations**	**Elasomeran (mRNA-1273)**	**Placebo**	**Relative (95% CI)**	**Absolute (95% CI)**		
**Clinical response - mRNA-1273**												
2	randomised trials	not serious	not serious	not serious	not serious	very strong association	66/28421 (0.2%)	929/28237 (3.3%)	**RR 0.07** (0.05 to 0.09)	**31 fewer per 1.000** (from 31 fewer to 30 fewer)	⊕⊕⊕⊕ High	CRITICAL
**AEs - mRNA-1273**												
8	randomised trials	not serious	very serious[a]	not serious	not serious	none	54353/60397 (90.0%)	27222/59944 (45.4%)	**RR 1.97** (1.95 to 1.99)	**441 more per 1.000** (from 431 more to 450 more)	⊕⊕◯◯ Low	IMPORTANT
**SAEs - mRNA-1273**												
4	randomised trials	not serious	not serious	not serious	serious[b]	none	608/30719 (2.0%)	650/30578 (2.1%)	**RR 0.93** (0.84 to 1.04)	**1 fewer per 1.000** (from 3 fewer to 1 more)	⊕⊕⊕◯ Moderate	CRITICAL

CI: confidence interval; **RR:** risk ratio

Explanations

a. Very high heterogeneity and p-value for heterogeneity; downgraded 2 steps for very serious inconsistency

b. Wide confidence intervals and crossed threshold; downgraded 1 step for serious imprecision
